# Investigating the Relationship between Stress and Emotional Intelligence with Academic Progress of Dental Students by Measuring Salivary Cortisol Levels 

**DOI:** 10.30476/dentjods.2025.104363.2526

**Published:** 2026-03-01

**Authors:** Yasamin Barakian, Fatemeh Kazemimoeen, Hamid Asayesh, Samira Hajisadeghi, Elham Keykha

**Affiliations:** 1 Dept. of Oral and Maxillofacial Medicine, School of Dentistry, Qom University of Medical Sciences, Qom, Iran.; 2 School of Dentistry, Qom University of Medical Sciences, Qom, Iran.; 3 Dept. of Medical Emergency, Qom University of Medical Sciences, Qom, Iran.; 4 Research Center for Prevention of Oral and Dental Diseases, School of Dentistry, Baqiyatallah University of Medical Sciences, Tehran, Iran.

**Keywords:** Emotional Intelligence, Stress, Cortisol, Saliva, Dental Student

## Abstract

**Background:**

Dental schools can be a stressful place that may impair students' performance. This stress can be related to several salivary biomarkers such as salivary cortisol. Also, emotional intelligence (EI) is one of the important factors in adapting people to the environment.

**Purpose:**

This research investigated the relationship between stress and EI with academic progress in Qom's Dental School students.

**Materials and Method:**

This cross-sectional (descriptive-analytical) study measured salivary cortisol using the enzyme-linked immunosorbent assay (ELISA) method. It involved 88 dental students in their third to sixth years. The required data were gathered using two standard questionnaires, namely Wang and Law’s EI scale and the dental environment stress questionnaire. Pearson's correlation coefficient and independent t-test were used for data analysis.

**Results:**

A total of 88 students participated in this research, of which 54.5% were female. Salivary cortisol had no relationship with EI or stress from the dental environment (*p* Value= 0.201). A direct relationship was found between EI and dental environment stress (*p* Value= 0.011).

**Conclusion:**

This study found no association between salivary cortisol levels, dental environment stress, and EI. Additionally, cortisol levels were inversely related to students' annual grade point average. Conversely, there was no correlation between annual grade point average and EI.

## Introduction

Medicine and dentistry are stressful academic fields that can harm students' performance. Dental students face many stressors while learning. They must master theories, clinical skills, and interpersonal skills.

Reports have shown that dental students are more stressed than those in other medical fields, and are more depressed and sensitive than their similar-age peers [ 1 - 4 ]. The stressful nature of dentistry starts from the very beginning because dental students must acquire a wide range of knowledge and skills to improve their education and careers [ 2 ].

Saliva is a good diagnostic fluid that is easy to collect, non-invasive, and more acceptable to patients than serum [ 5 ]. Saliva's composition is affected by many factors, including short-term, acute psychological stress. Also, many salivary markers are related to stress including cortisol, alpha-amylase, beta-endorphin, and chromogranin-A [ 6 ]. Chronic stress is related to the activity of the hypothalamus-pituitary-adrenal (HPA) axis and is measured by salivary cortisol. Long-term activation of this axis is harmful to the health of organs. The correlation between salivary cortisol and plasma cortisol is greater than 0.9 [ 6 ]. Thus, salivary cortisol can be considered as its equivalent in serum. Researchers consider salivary cortisol concentration a reliable marker for stress [ 7 ]. Recent research has shown that cognitive intelligence cannot alone determine success. Other factors, including emotional intelligence (EI), are also involved [ 8-10 ]. EI is "the ability to recognize one's own and others' emotions, motivate oneself, and manage emotions well in ourselves and our relationships" [ 11 ]. This intelligence is related to a person's knowledge of himself and others and includes communication, compatibility, and adapting to the environment. These skills are vital for meeting social demands. They are also a tactical asset for individual performance [ 12 - 13 ].

Previous studies found that EI boosts academic progress. This finding highlights its importance in education, work, social life, and mental health. In this respect, EI is crucial for higher academic performance in clinical courses [ 13 - 16 ].

Academic progress is among the most important criteria that play a significant role in assessing the ability of students to complete their university education and reach graduation. This concept is one of the most important parameters used to predict the future status of learners in terms of acquiring the necessary scientific and practical qualifications and skills [ 17 ]. Academic progress is typically measured in various ways, such as the level of progress in each educational course separately, the progress achieved in educational courses, the annual grade point average (GPA), and the GPA of an educational program. GPA is one of the common indicators used to evaluate academic performance. Many faculties consider the minimum level as a criterion for passing students' exams [ 18 - 20 ]. Investigating the effect of stress on academic progress has shown that stress among dental students, especially during clinical training, can be a serious threat that can harm students' performance and the profession of dentistry [ 21 - 22 ]. Besides, it suggested that students' EI can influence practical outcomes. Interventions to enhance EI can be developed to improve patient satisfaction [ 23 ]. Therefore, the present research was conducted to study the relationship between stress (by measuring the salivary cortisol level and using a standard questionnaire) and EI with academic progress. 

## Materials and Method

This cross-sectional (descriptive-analytical) study was approved by the Ethics Committee of Qom University of Medical Sciences with the code IR.MUQ.REC.1399. 186. The research population included 88 students (48 female and 40 male students) from the 3rd to 6th year, between 21 and 27 years old, at Qom University of Medical Sciences' dentistry faculty. They had no systemic disease and were not taking medication. The sample size of 85 people was calculated based on the correlation coefficient between stress and cortisol levels in Pani's study [ 24 ]. To account for possible attrition, 10% was added to the sample size. In the end, 88 students were included in the study. (α= 0.05, β= 0.2, r= 0.3, The standard normal deviate for α= Zα= 1.960, The standard normal deviate for β= Zβ= 0.842, C= 0.5 * ln = 0.310, Total sample size= N = 2 + 3 =85)

A consent form was obtained from the participants to evaluate the students' academic progress using their GPA from the last two years. Then chronic stress level was evaluated by measuring salivary cortisol. Researchers collected the data by using a standard dental environment stress (DES) questionnaire [ 25 ]. This questionnaire contains 37 questions in 6 fields including academic efficiency (5), treatment problems (5), self-evaluation (5), personal and family factors (8), clinical education (11), and other cases (3). Any question uses a 4- point Likert scale: 1= never, 2= slightly, 3= moderate, 4= severe. The validity index of this questionnaire was 79% (25). Besides, its reliability coefficient was determined by Cronbach's alpha test in each of the areas of academic efficiency: 84%, treatment problems: 78%, self-evaluation: 82%, personal, and family factors: 79%, clinical education: 81%, and other items: 77% [ 25 ].

Also, for each field, the average stress score of its items was considered. As a result, the DES score ranges from 32 to 128. The ranges for other fields are: academic efficiency, 7-28; treatment problems, 4-16; self-evaluation, 4-16; personal and family factors, 2-8; clinical education, 11-44; and other cases, 4-16. 

Students' EI was assessed using Wang and Lau's questionnaire, which has 16 questions. It includes sections on self-emotion appraisal (4 questions), others’ emotion appraisal (4 questions), use of emotion (4 questions), and regulation of emotion (4 questions), ranging from 1 (strongly disagree) to 7 (strongly agree) for any question. Higher scores indicate a higher level of EI. 

This standard questionnaire is valid and reliable, and the Cronbach's alpha coefficient is r=0.081 [ 26 ]. 

To measure salivary cortisol, we rested each person for 20 to 40 minutes after the questionnaires. Then, we collected their unstimulated saliva. In this research, it was tried to avoid circadian rhythm changes by collecting all samples using the spitting method [ 27 ]. They were collected within a set time range (9 to 11 A.M). The participants refrained from exercising, eating, smoking, drinking, or any oral stimulation, such as using mouthwash and brushing teeth, for 90 minutes before collecting saliva. They also avoided talking and any head movements while collecting saliva [ 28 - 30 ]. After rinsing the mouth with water and swallowing, the subjects had 5 minutes to collect their saliva and pour it into a special plastic tube (Falcon). After the start of the time, the participant tilted their heads down (45 ° angle) and spited saliva into the falcon tube 1-2 times a minute until 5 minutes were up [ 28 ]. It was possible to freeze saliva before measuring relevant factors. Due to the time-consuming nature of collecting samples, saliva samples were frozen at -20oC from collection until the test was performed [ 5 ]. Cortisol measurement was also done using the relevant kits from DiaMetra company (made in Italy) and the enzyme-linked immunosorbent assay (ELISA) method. According to the kit catalog, a normal range for AM cortisol is 3-10 ng/mL, less than 4.8 is considered low, 4.8-9.5 is considered moderate, and more than 9.5 is considered high. Data were expressed as mean and standard deviation (±SD) and number (%) for quantitative and qualitative variables, respectively. In this research, Pearson's correlation coefficient and an independent t-test were used to analyze the data. Also, a p Value of less than 0.05 was considered to be statistically significant. 

## Results

Eighty-eight dental students (48 women and 40 men) participated in this research. The average age of the participants was 22.77 years (±2.93) and 22 of them were in third year, 28 in fourth year, 23 in fifth year, and 15 in sixth year. Considering EI levels, 23 students (26.1%) had a low level (poor; < score 25), 45 students (51.1%) had a medium level (score 25-37), and 20 students (22.7%) had a high level (good; > score 37). Regarding DES questionnaire, 24 students (27.3%) reported a low level, 42 students (47.7%) reported a moderate level, and 22 students (25.0%) reported a high level.

Salivary cortisol levels were observed to be low in 23 students (26.1%), moderate in 43 students (48.9%), and high in 22 students (25.0%). Notably, fourth-year students exhibited the highest cortisol levels, while sixth-year students had the lowest.

The total score of EI was 32.78±6.42. This score included a self-emotion appraisal score of 8.15±2.24, an others’ emotion appraisal at 9.35±2.51, a score of 7.87± 2.40 for the use of emotion, and 7.40±1.87 for regulation of emotion. Notably, third-year students had the highest EI score at 33.42±7.06, followed by sixth-year students at 33.00±5.64, fifth-year students also at 33.00 ±5.33, and fourth-year students at 32.00±7.33.

The average score for students regarding DES questionnaire was 97.12±24.35. This score included scores of 13.98±4.30 in the self-evaluation section, 18.00±6.16 in the personal and family factors section, 15.67±4.47 in the academic efficiency section, 28.84±7.84 in the clinical education section, and 13.22±4.66 in the treatment problems section. Notably, sixth-year students recorded the highest DES scores.

The mean GPA of students was 15.92±1.91, which increased to 16.49±1.73 the following year. The highest GPA was recorded among sixth-year students.

The average cortisol level of the students was 7.93± 3.91. No correlation was identified between cortisol levels and DES or its total score (*p*= 0.205). Additionally, there was no correlation between cortisol levels and EI or its total score (*p*= 0.231). 

There was no significant difference in the average cortisol level and GPA between male and female students. Also, the total score of DES was not significantly different between the two groups. However, the total score for EI was significantly higher among female students than male students.

Table 1 shows several direct relationships. First, there was a direct relationship between the DES total score and the total EI score. This includes self-emotion appraisal, use of emotion, and others’ emotion appraisal. 

**Table 1:** The relationship between dental environment stress (DSE) and emotional intelligence (EI)

Variable		The total score of EI	self-emotion appraisal	Regulation of emotion	Use of emotion	Others’ emotion appraisal
Total score of DSE	Pearson Correlation	.271	.296	.006	.214	.219
	*p* Value	.011*	.005*	.953	.045*	.042*
self-evaluation	Pearson Correlation	.209	.293	-.034	.228	.076
	*p* Value	.051	.006*	.756	.032*	.486
personal and family factors	Pearson Correlation	.210	.191	.075	.105	.208
	*p* Value	.050	.074	.489	.332	.053
Academic efficiency	Pearson Correlation	.247	.246	.000	.151	.271
	*p* Value	.020*	.021*	.997	.161	.011*
Clinical education	Pearson Correlation	.317	.343	.023	.264	.237
	*p* Value	.003*	.001*	.830	.013*	.027*
treatment problems	Pearson Correlation	.084	.110	-.066	.110	.069
	*p* Value	.438	.309	.541	.307	.527

Second, the total score of EI had a direct relationship with academic efficiency and clinical education. Moreover, there was a direct relationship between self-evaluation, self-emotion appraisal, and applying emotions. Similarly, academic efficiency was related to self-emotional appraisal and others’ emotion appraisal. Lastly, clinical education was significantly associated with self-emotion appraisal, the use of emotion, and others’ emotional appraisal. 

According to Table 2, cortisol levels had an inverse relationship with GPA and a direct relationship with age (*r*= 0.34, *p*= 0.000). 

**Table 2:** Relationship between cortisol , dental environment stress (DES), academic progress (annual grade point average (GPA)) and emotional intelligence (EI)

Variable		Academic year	Cortisol levels	GPA of 2020	GPA of 2021	Age	Total DES score	Total EI score
Academic year	Pearson Correlation		-0.02	-0.10	0.21	0.36	0.09	-0.00
	*p* Value		0.891	0.352	0.051	0.000*	0.402	0.972
Cortisol levels	Pearson Correlation	-0.02		-0.26	-0.22	0.34	-0.13	0.14
	*p* Value	0.891		0.026*	0.042*	0.000*	0.235	0.202
GPA of 2020	Pearson Correlation	-0.10	-0.26		0.85	-0.21	-0.04	-0.13
	*p* Value	0.352	0.026*		0.000*	0.052	0.710	0.230
GPA of 2021	Pearson Correlation	0.21	-0.22	0.85		-0.03	-0.04	-0.09
	*p* Value	0.051	0.042*	0.000*		0.814	0.751	0.423
Age	Pearson Correlation	0.36	0.34	-0.21	-0.03		-0.08	-0.19
	*p* Value	0.000*	0.000*	0.052	0.814		0.477	0.082
Total DES score	Pearson Correlation	0.09	-0.13	-0.04	-0.04	-0.08		0.27
	*p* Value	0.402	0.235	0.710	0.751	0.477		0.011*
Total EI score	Pearson Correlation	-0.00	0.14	-0.13	-0.09	-0.19	0.27	
	*p* Value	0.972	0.202	0.230	0.423	0.082	0.011*	

## Discussion

The research findings showed that dental students are exposed to many stressful factors [ 1 , 31 ] In addition, the findings of a review study showed that EI plays a greater role in the academic success of medical and dental clinical year students [ 32 ]. A statistically significant relationship between EI and academic success was reported, which is essential for improving academic performance in dental education [ 33 ]. Therefore, this study aimed to investigate the relationship between stress and EI with academic performance. In our study, the student's score on the DES questionnaire was 97.12±24.35 (out of 165). This number was similar to the studies of Dalband *et al*. [ 34 ] (96.20±20.35) and Ramezani *et al*. [ 25 ] (88.06±16.28). In the present study, there was no significant relationship between stress (as measured by the DES questionnaire) and the academic year. Our finding is consistent with the results of Ramezani *et al*. [ 25 ] study. Several studies found the highest level of stress in fourth-year students [ 34 - 37 ]. In Sangiorgio *et al*. [ 38 ] study, stress was highest among fifth-year students compared to 4th and 3th-year students. High stress among fourth and fifth year students has been attributed to the large number of theoretical and practical courses in addition to entering clinical work and starting practice on patients [ 15 , 36 ]. The difference between the results of our study and these studies may be due to the course plans, professor-student interactions, and differing university rules in various regions, such as outside work.

In the present study, there was no significant relationship between stress, as measured by the DES questionnaire, and gender. Although in several studies fema- students had a higher stress level [ 25 , 34 - 35 , 38 - 39 ]. Tangade *et al*. [ 40 ] study showed that men feel more stress than women. Our study found that, unlike other studies, female students were not more stressed than male students. Women have the same level of stress as men in the dental environment, probably because of the support from professors and a good educational environment.

In our study, there was no correlation between stress based on the score of the DES questionnaire and the salivary cortisol level. The results of the present study were consistent with those of Pani *et al*. [ 24 ] study. In Ng *et al*. [ 41 ] study, students had more stress and higher salivary cortisol levels before exam. This difference between the results of studies may be due to the varying sampling times in both studies. Moreover, DES may occur only during dental procedures Thus, it cannot be accurately measured outside of them.

Based on Wang and Lau's EI questionnaire in this study, the average scores of students in EI were low (32.81±6.39) (out of 64 scores). Binandeh *et al*. [ 42 ] found a weak EI (221.39 out of 450) for students. Our results were somewhat similar to their results. In two studies conducted by Ehsani *et al*. [ 43 ] (using Bar-An questionnaire, 353.49 out of 450 scores) and Saddki *et al*. [ 44 ] (assessing emotions scale, 121.2 out of 165 scores), moderate EI was reported in students. Also, in a comparative study of EI through the Bar-An questionnaire in male and female students, most students (69.23%) scored high [ 45 ]. In another study of the EI of medical and dental students through the Bar-An questionnaire, the overall mean of this score among all students (331.16 out of 450 scores) and specifically in dental students (326.2 out of 450 scores) was reported to be good [ 46 ]. The explanation for the variability of these reports might be the use of different questionnaires and statistical population. However, this study found the highest EI score was related to others’ emotional appraisal. The lowest was related to the regulation of emotion. In Ehsani *et al*. [ 43 ] study, the lowest average was in stress tolerance and the highest was in empathy. Bahrami *et al*. [ 45 ] study found that girls had the highest mean in stress control while boys had the lowest in adaptation. Also, in a study the average emotion regulation was lower than other components [ 46 ]. Another study reported that empathy and emotion regulation may not be significantly related in dental students [ 47 ].

The total score of EI was related to gender. Female students scored higher than male students (P: 0.01). However, this finding was inconsistent with a few studies [ 13 , 48 - 49 ] and consistent with several studies [ 42 - 45 , 47 , 50 - 52 ]. A review of studies on gender and EI showed that EI is a flexible skill that can be improved by our life activities [ 53 ]. Expectations vary based on gender. Culturally, girls are encouraged to express feelings, but boys are often taught to hide their emotions. This result supports a tough, masculine image. Studies show that diversity, sample sizes, and questionnaires all play a role. Additionally, local customs and cultures have an impact [ 42 , 46 , 54 ].

No correlation was observed between the salivary cortisol level EI components, and its total score. Mikolajchak *et al*. [ 55 ] study showed a different result, reporting that people with higher EI had lower salivary cortisol levels when stressed. Another study found a significant difference between general and EI. General intelligence does not relate to acute or chronic stress levels. In contrast, high EI is related to lower levels of both types of stress. This data suggests that EI helps people better manage both acute and chronic daily stress [ 56 ]. High salivary cortisol levels enhance coping strategies in EI, especially during acute stress. Pau *et al*. [ 37 ] study also found that EI helps dental students manage stress contradicting our study. Accordingly, we suggest a larger study to explore the link between EI and salivary cortisol levels, a stress biomarker, particularly in medical students. 

The study found no relationship between salivary cortisol with DES and EI. It found a direct relationship between DES and EI. In other words, more EI means more stress in the dental environment. The explanation for this contradictory result is that the study was conducted during exams course. Hence, larger studies are needed to avoid Fishing Bias. Also, examining the dental environment's impact on EI and stress is crucial. Such a study should include factors like academic failure and awareness of stress-coping methods among students. A suitable sample size is essential for these studies. 

Cortisol level (stress) had an inverse relationship with GPA and a direct relationship with age. This result suggests that students with higher GPAs tend to have lower salivary cortisol levels, while older individuals exhibit higher cortisol levels. Also, GPA in 2021 was significantly higher than that of 2020. We can reduce this stress by modifying the dental curriculum. We should add stress prevention and intervention programs from the first year. 

## Conclusion

This study found no association between salivary cortisol levels, DES, and EI. However, there was a direct relationship between DES and EI. Additionally, cortisol levels were inversely related to students' GPAs. This finding means higher GPAs matched with lower cortisol levels, indicating less stress. Conversely, there was no correlation between GPA and EI. 

## References

[1] Rizvi F, Qureshi A, Rajput AM, Afzal M ( 2015). Prevalence of depression, anxiety and stress (by DASS scoring system) among medical students in Islamabad, Pakistan. Br J Med Med Res.

[2] Polychronopoulou A, Divaris K ( 2009). Dental students’ perceived sources of stress: a multi‐country study. J Dent Educ.

[3] Shehada MR, Alfakhry G, Jamous I, Aljoujou AA, Abdul-Hak M ( 2023). Major stress sources amongst dental students at Damascus University, Syria. Int Dent J.

[4] de Souza Ferreira F, Barros I, da Costa Neves T, Pazos JM, Garcia PPNS ( 2023). Stress amongst dental students in the transition from preclinical training to clinical training: A qualitative study. Eur J Dent Educ.

[5] Soo-Quee Koh D, Choon-Huat Koh G ( 2007). The use of salivary biomarkers in occupational and environmental medicine. Occup Environ Med.

[6] Kirschbaum C, Hellhammer DH ( 1989). Salivary cortisol in psychobiological research: an overview. Neuropsychobology.

[7] Clow A ( 2004). Cortisol as a biomarker of stress. J Holistic Healthcare.

[8] Cho S ( 2010). The role of IQ in the use of cognitive strategies to learn information from a map. Learning Individual Differences.

[9] Wong CS, Law KS (2002). The effects of leader and follower emotional intelligence on performance and attitude: An exploratory study. Leadership Quarterly.

[10] Lazin R, Neumann L ( 1991). Student characteristics as predictors of drop‐out from medical school: admissions to beersheva over a decade. Med Educ.

[11] Emmerling RJ, Goleman D ( 2003). Emotional intelligence: Issues and common misunderstandings. Issues Recent Developments Emotional Intelligence.

[12] Radafshar G, Zarrabi H, Jalayer S ( 2012). Relationships of Stress and Coping Styles to Periodontal Disease: A Case-Control Study. J Dent Shiraz Univ Med Sci.

[13] Ghofrani Kelishami F, Farahani M, Jamshidi Orak R, Seyedfatemi N, Banihashemi S ( 2015). Investigation the Relationship between Emotional Intelligence and Demographic Information of Nursing Students of Tehran University of Medical Sciences. Iranian J Nursing Res.

[14] Victoroff KZ, Boyatzis RE (2013). What is the relationship between emotional intelligence and dental student clinical performance?. J Dent Educ.

[15] Jahan SS, Nerali JT, Parsa AD, Kabir R ( 2022). Exploring the Association between Emotional Intelligence and Academic Performance and Stress Factors among Dental Students: A Scoping Review. Dent J.

[16] Haralur S, Majeed M, Afzal M, Chaturvedi S ( 2019). Association of sociodemographic factors and emotional intelligence with academic performance in clinical and preclinical dental courses. Nigerian J Clin Prac.

[17] Soares AP, Guisande AM, Almeida LS, Páramo FM ( 2009). Academic achievement in first‐year Portuguese college students: The role of academic preparation and learning strategies. Int J Psychol.

[18] Pitt V, Powis D, Levett-Jones T, Hunter S ( 2012). Factors influencing nursing students' academic and clinical performance and attrition: An integrative literature review. Nurse Educ Today.

[19] Hasegawa Y, Ninomiya K, Fujii K, Sekimoto T ( 2016). Emotioonal intelligence score and performance of dental undergraduates. Odontology.

[20] Fernandez R, Salamonson Y, Griffiths R ( 2012). Emotional intelligence as a predictor of academic performance in first‐year accelerated graduate entry nursing students. J Clin Nursing.

[21] Alzahem AM, Van der Molen  HT, De Boer  BJ (2013). Effect of year of study on stress levels in male undergraduate dental students. Adv Med Educ Pract.

[22] Al-Saleh SA, Al-Madi EM, Al-Angari NS, Al-Shehri HA, Shukri MM ( 2010). Survey of perceived stress-inducing problems among dental students, Saudi Arabia. Saudi Dent J.

[23] Mohan M, Lin KH, Parolia A, Pau A (2021). Does Emotional Intelligence of Dental Undergraduates Influence Their Patient Satisfaction?. Int J Dent.

[24] Pani SC, Al Askar AM, Al Mohrij SI, Al Ohali TA ( 2011). Evaluation of stress in final‐year Saudi dental students using salivary cortisol as a biomarker. J Dent Educ.

[25] Ramazani N, Nazari A ( 2013). Dental environmental stress among clinical dentistry students in Zahedan School of Dentistry. Iranian J Med Educ.

[26] Law KS, Wong CS, Song LJ ( 2004). The construct and criterion validity of emotional intelligence and its potential utility for management studies. J Appl Psychol.

[27] Navazesh M ( 1993). Methods for collecting saliva. Ann N Y Acad Sci.

[28] Arhakis A, Karagiannis V, Kalfas S ( 2013). Salivary alpha-amylase activity and salivary flow rate in young adults. Open Dent J.

[29] Roberts J ( 2018). How Manuscripts Are Evaluated at Headache: The Journal of Head and Face Pain. Headache.

[30] Bugdayci G, Yildiz S, Altunrende B, Yildiz N, Alkoy S ( 2010). Salivary alpha amylase activity in migraine patients. Auton Neurosci.

[31] Rada RE, Johnson-Leong C ( 2004). Stress, burnout, anxiety and depression among dentists. J Am Dent Assoc.

[32] Singh N, Kulkarni S, Gupta R ( 2020). Is emotional intelligence related to objective parameters of academic performance in medical, dental, and nursing students: A systematic review. Educ Health.

[33] Alsaif MI, Aljuni A, Alyemni K, Almuntashiri F, Hamdan HM, Alamri H, et al ( 2024). The Association between Emotional Intelligence and Academic Performance of Dental Students at King Saud University, Riyadh, Saudi Arabia. Cureus.

[34] Dalband M, Farhadi Nasab A ( 2007). Evaluation of stress-inducing factors of educational environment in Hamadan Dentistry School’s students. Avicenna J Clin Med.

[35] Mahdizadeh M, Kheirkhah F, Vojdani FH, Noori BS ( 2014). Stress factors in dental students of Babol University. J Dent Sch.

[36] Akbari M, Nejat A, Dastorani S, Rouhani A ( 2011). Evaluation of stress level and related factors among students of Mashhad Dental School (Iran) in academic year of 2008-2009. J Mashhad Dent Sch.

[37] Pau A, Croucher R, Sohanpal R, Muirhead V, Seymour K ( 2004). Emotional intelligence and stress coping in dental undergraduates- a qualitative study. British Dent J.

[38] Sangiorgio JPM, Araujo PM, Navarro CH, Zen IR, da Costa SC, Ribeiro PHV, et al ( 2016). Dental environment stress: Findings among Lusophone dental students. Brazilian Res Ped Dent Integrat Clinc.

[39] Shahravan A, Karimi-Afshar M, Torabi M, Safari S ( 2016). Assessment of dental environment stress among clinical dentistry students in Kerman dental school Iran in 2014. Strides Develop Med Educ J.

[40] Tangade PS, Mathur A, Gupta R, Chaudhary S ( 2011). Assessment of stress level among dental school students: an Indian outlook. Dent Res J.

[41] Ng V, Koh D, Mok BY, Chia SE, Lim LP ( 2003). Salivary biomarkers associated with academic assessment stress among dental undergraduates. J Dent Educ.

[42] Binandeh ES, Hosseinpour S, Binandeh ES, Jabbarifar SE (2019). Investigation of the Relationship between the Emotional Intelligence and Academic Performance of Undergraduate Students of Isfahan Dental School. J Isfahan Dent Sch.

[43] Ehsani M, Sabarad L, Kheirkhah F ( 2014). The relationship between emotional intelligence and academic achievement in the dental students of Babol medical university. Educational Development Judishapur.

[44] Saddki N, Sukerman N, Mohamad D ( 2017). Association between emotional intelligence and perceived stress in under graduate dental students. Malaysian J Med Sci (MJMS).

[45] Bahrami N, Najafvand Derikvandi  S, Bahrami S ( 2017). The comparison of Emotional Intelligence among University Students at Dezful Medical University. Educational Development Judishapur.

[46] Ahmadi S, Baradaran TR, Hosseini M, Mehrabian F ( 2014). The study of emotional intelligence in medical & dentistry students of international branch of Guilan University of Medical Sciences in 2013. Res Med Edu.

[47] Lermen C, Wetzel W, Britz V, Sterz J, Bechstein WO, Schreckenbach T ( 2022). Empathy, personality traits, and emotional management in 2nd and 4th-year dentistry students: a single-center study. BMC Med Educ.

[48] Bishr EM, Amer HA, Saleh SM ( 2016). Emotional intelliigence among alexandria university dental interns. Alexandria Dent J.

[49] Sa B, Ojeh N, Majumder MAA, Nunes P, Williams S, Rao SR, Youssef FF ( 2019). The relationship between self-esteem, emotional intelligence, and empathy among students from six health professional programs. Teach Learn Med.

[50] Niazi A, Qayyum M, Ikram Z, Sher S, Sethi MR, Irfan M ( 2019). Emotional Intelligence and Self-Perception of Medical and Dental Students of Peshawar–Pakistan. J Postgraduate Med Institute.

[51] Partido BB, Stefanik D ( 2020). Impact of emotional intelligence training in a communication and ethics course among second‐year dental students. J Dent Educ.

[52] Partido BB, Stafford R ( 2018). Association between emotional intelligence and academic performance among dental hygiene students. J Dent Educ.

[53] Naghavi F, Redzuan M ( 2011). The relationship between gender and emotional intelligence. World Appl Sci J.

[54] Extremera N, Fernández-Berrocal P, Salovey P (0: reliabilities, age and gender differences Psicothema 2006). Spanish version of the Mayer-Salovey-Caruso Emotional Intelligence Test (MSCEIT). Version 2.

[55] Mikolajczak M, Roy E, Luminet O, Fillée C, De Timary  P ( 2007). The moderating impact of emotional intelligence on free cortisol responses to stress. Psychoneuroendocrinology.

[56] Singh Y, Sharma R ( 2012). Relationship between general intelligence, emotional intelligence, stress levels and stress reactivity. Ann Neurosci.

